# Self-compassion and its barriers: predicting outcomes from inpatient and residential eating disorders treatment

**DOI:** 10.1186/s40337-022-00640-8

**Published:** 2022-08-06

**Authors:** Josie Geller, Lindsay Samson, Nadia Maiolino, Megumi M. Iyar, Allison C. Kelly, Suja Srikameswaran

**Affiliations:** 1 Eating Disorders Program, St. Paul’s Hospital, 1081 Burrard Street, Vancouver, BC V6Z 1Y6 Canada; 2 Department of Psychiatry, University of British Columbia, Vancouver, Canada; 3 Vancouver Coastal Health, Vancouver, Canada; 4 Department of Psychology, University of British Columbia, Kelowna, Canada; 5 Department of Psychology, University of Waterloo, Waterloo, ON Canada

**Keywords:** Self-compassion, Fear of self-compassion, Barriers to self-compassion, Eating disorders, Treatment

## Abstract

**Background:**

Individuals with eating disorders (EDs) experience barriers to self-compassion, with two recently identified in this population: Meeting Standards, or concerns that self-compassion would result in showing flaws or lead to loss of achievements or relationships, and Emotional Vulnerability, or concerns that self-compassion would elicit difficult emotions such as grief or anger. This exploratory study examined the utility of self-compassion and two barriers to self-compassion in predicting clinical outcomes in intensive ED treatments.

**Method:**

Individuals in inpatient (*n* = 87) and residential (*n* = 68) treatment completed measures of self-compassion and fears of self-compassion, and ten clinical outcome variables at pre- and post-treatment.

**Results:**

Pre-treatment self-compassion was generally not associated with outcomes, whereas pre-treatment self-compassion barriers generally were. In both treatment settings, fewer Emotional Vulnerability barriers were associated with improved interpersonal/affective functioning and quality of life, and fewer Meeting Standards barriers were associated with improved readiness and motivation. Interestingly, whereas Meeting Standards barriers were associated with less ED symptom improvement in inpatient treatment, Emotional Vulnerability barriers were associated with less ED symptom improvement in residential treatment.

**Conclusions:**

Given that few longitudinal predictors of outcome have been established, the finding that pre-treatment barriers to self-compassion predict outcomes in both inpatient and residential settings is noteworthy. Targeting self-compassion barriers early in treatment may be helpful in facilitating ED recovery.

## Plain English summary

Self-compassion is associated with a number of positive health indicators and has been shown to support recovery from an eating disorder. This exploratory study examined whether self-compassion and barriers to self-compassion predict clinical outcomes in inpatient and residential eating disorders treatment settings. Whereas self-compassion was rarely associated with outcome, self-compassion barriers were associated with the majority of variables investigated. Given that few longitudinal predictors have been established in this field, the relation between barriers to self-compassion and outcome in both inpatient and residential settings is noteworthy. Targeting self-compassion barriers early in treatment may be helpful in facilitating eating disorder recovery.

## Introduction

Self-compassion refers to being sensitive to our suffering and motivated to alleviate it [1]. It has been operationalized as responding to personal suffering with mindfulness, a recognition of our common humanity, and self-kindness [2]. Self-compassion has been associated with a wide range of positive health indicators, including better relationships, more pro-active health behaviours, and resilience in times of difficulty [3, 4]. Despite a robust literature showing its association with positive well-being, many individuals have barriers to practicing self-compassion, as described by Gilbert and colleagues and operationalized in the Fears of Compassion for Self scale [5].

Individuals with eating disorders (EDs) have lower levels of self-compassion and higher levels of fear of self-compassion relative to non-clinical samples [6]. These self-compassion fears, which we refer to as barriers to self-compassion, are associated with higher ED and psychiatric symptomatology, poorer intra- and interpersonal functioning, and lower quality of life [7–9]. Individuals who have received ED treatment describe developing self-compassion as central to their recovery [10, 11], with greater gains in self-compassion early in treatment predicting decreases in shame over 12 weeks of treatment [12]. In encouraging individuals to experience difficult emotions with kindness and without judgment, self-compassion may reduce distress, facilitate self-acceptance, and decrease the need to engage in ED behaviours. Indeed, preliminary evidence suggests that overcoming barriers to self-compassion is related to the benefits individuals with EDs experience from treatment. For instance, Kelly and colleagues [9] demonstrated that a combination of lower self-compassion and higher fear of self-compassion at baseline was associated with no improvement in ED symptoms in a combined sample of individuals undergoing inpatient or day therapy (*N* = 74).

A recent factor analysis of the Fears of Compassion for Self scale in individuals with EDs indicated that there are two types of barriers to developing self-compassion in this population: Meeting Standards, or concerns that cultivating self-compassion would lead one to show their flaws and lose achievements and relationships, and Emotional Vulnerability, or concerns that self-compassion would involve experiencing difficult emotions such as grief, anger and/or hurt [8]. A recent cross-sectional examination of individuals with EDs (*N* = 349) indicated that the two barriers to self-compassion were significantly associated with variables of clinical interest. Specifically, Meeting Standards barriers were associated with a more rigid thinking style and lower readiness for change, whereas Emotional Vulnerability barriers were associated with ED and psychiatric symptom severity, lower self-compassion, intra- and interpersonal functioning difficulties, lower readiness and motivation to change, and impaired quality of life [7].

The identification of self-compassion as a marker of health and the demonstrated relations among self-compassion, barriers to self-compassion and ED symptomatology [8, 9] suggest that self-compassion variables may play a role in patients’ responsiveness to treatment. A comprehensive review of variables associated with clinical outcome in individuals with EDs found that aside from symptom severity, readiness and motivation for behavioural change was the only consistent predictor of outcome [13, 14]. This awareness of the clinical importance of readiness and motivation for behavioural change led to improvements in evidence-based therapies. Specifically, adapted treatments such as enhanced cognitive behaviour therapy [15] have come to routinely assess and address readiness, and empirically-based guidelines have been developed that use readiness information to allocate treatment based on patient needs [16]. If self-compassion variables were identified as playing a role in treatment outcome, interventions could be similarly improved by directly targeting these variables. In fact, barriers to self-compassion may be thought of as a form of readiness, or one’s current capacity for developing self-compassion.

The current study builds upon existing research in two ways. First, with the recent identification of two distinct types of barriers to self-compassion in people with eating EDs, this research examined the unique contribution of these two barrier types to clinical outcome in patients receiving ED treatment. Second, sample size constraints in previous research may have precluded the ability to separately examine outcomes in inpatient and day therapy. Given that inpatient and day therapy/residential care differ in treatment goals (medical stabilization vs. full recovery) and patient profiles (higher symptom severity, lower readiness for change vs. lower symptom severity, higher readiness for change), there is a need to examine the role of self-compassion variables in each of these samples. The purpose of this exploratory study was to examine whether self-compassion and the two barriers to self-compassion were associated with clinical outcome. Although this research is exploratory in nature, it was hypothesized that higher pre-treatment self-compassion scores and lower pre-treatment barriers to self-compassion scores would be associated with greater benefit from inpatient and residential treatment.

## Methods

### Participants

Patients referred to a specialized ED treatment program within a large metropolitan Canadian hospital (*N* = 155) were recruited to participate in this research. Participants included patients from an inpatient program (*n* = 87) and a residential treatment program (*n* = 68).

In the inpatient program, the mean age of participants was 32.84 years (*SD* = 12.34). Eighty-one (93%) participants identified as female, two (2%) as male, and four (5%) as transgender. Sixty-nine (78%) individuals identified as Caucasian, three (3%) as East Asian, two as Hispanic/Latino (2%), two (2%) as mixed race, two (2%) as Indigenous, one (1%) as South Asian, and ethnicity was not available for eight (9%) individuals. Mean illness duration was 16.34 years (*SD* = 13.24). Diagnoses were assigned by the program psychiatrist following a semi-structured clinical interview. Forty-seven (53%) participants received a diagnosis of anorexia nervosa (AN), 27 (31%) were diagnosed with bulimia nervosa (BN), and 13 (15%) were diagnosed with other specified feeding or eating disorder (OSFED). Mean body mass index (BMI) at admission for those with AN was 15.96 kg/m^2^ (*SD* = 2.08) and for the remainder of the sample (BN and OSFED) was 23.37 kg/m^2^ (SD = 7.03).

In the residential treatment program, the mean age of study participants was 32.81 years (*SD* = 9.78). Sixty-five (96%) participants identified as female and three (4%) as male. Fifty-four individuals (79%) identified as Caucasian, four as East Asian (6%), eight as mixed race (12%), one (2%) as Indigenous, and ethnicity was not available for one individual. Mean illness duration was 17.86 years (*SD* = 12.97). Twenty-one (31%) participants were diagnosed with AN, 31 (46%) were diagnosed with BN, 15 (22%) with OSFED, and diagnosis was not available for one individual. Mean body mass index (BMI) at admission for those with AN was 19.37 kg/m^2^ (*SD* = 2.71) and for the remainder of the sample (BN and OSFED) was 25.89 kg/m^2^ (*SD* = 7.17).

### Procedure

#### Inpatient treatment

The goals of the inpatient treatment program were medical stabilization and/or symptom interruption, and improvements in readiness and motivation for change. Patients admitted to this level of care had a high level of symptom severity (e.g., severe dietary restraint, binge eating or purging multiple times per day), and a moderate to high level of medical acuity (e.g., electrolyte or cardiac abnormalities and rapid weight loss).

In addition to medical care, patients received meal support to help them follow their prescribed meal plan. Four psychotherapy groups were offered per week, and patients received two to three individual sessions per week with their psychiatrist, dietitian and social worker. The majority of groups were adjunctive in nature (e.g., leisure, spiritual care, self-care, yoga), due to the high medical severity and low readiness of the population. Patients received three to five psychoeducation sessions that addressed self-compassion skills. All admissions were voluntary and ranged in length from five to 83 days, with a mean length of stay of 43.45 days.

#### Residential treatment

The goal of the residential treatment program was full recovery, focusing on developing adaptive coping strategies, building greater awareness around the function of the ED, and increasing understanding of underlying issues (e.g., trauma). Patients admitted for residential treatment were medically stable but had moderate to high levels of symptom severity, though less severe than those admitted to inpatient treatment. Residential patients also demonstrated higher levels of readiness/engagement to change their ED than did the inpatient population (e.g., willing and able to eat recommended meal plans, refrain from bingeing or use of compensatory strategies, and meeting weight gain targets).

Residential patients received meal support and group and individual therapy. Ten psychotherapy groups were offered per week, and patients received three to four individual sessions per week with their psychologist, dietitian, family therapist and support worker. Topics covered in skills groups included assertiveness, body image, and self-compassion, while process groups provided the opportunity to explore issues underlying the development and maintenance of the ED and the impact of these issues on relations with self and others. Length of stay in residential treatment ranged from 12 to 15 weeks, with a mean of 101.85 days.

Patients referred to these programs were eligible and invited to participate in research. At pre- and post-treatment for each program, a research assistant met with each patient individually to administer a research battery consisting of outcome measures, namely: ED symptoms, interpersonal and affective functioning, readiness and motivation for change, and quality of life. At pre-treatment, participants also completed the Self-Compassion Scale and the Fears of Compassion for Self scale. Ethics approval was obtained for both the data collection and methodology for this study.

### Measures

#### Self-Compassion Scale (SCS; [2])

This 26-item measure provides a total self-compassion score, which was used in this research. Examples of items include ‘I try to be understanding and patient toward aspects of my personality I don’t like’ and ‘When something painful happens I try to take a balanced view of the situation.’ The SCS has demonstrated good internal consistency reliability, as well as good test–retest reliability over a 3-week interval [2]. The internal consistency of the SCS was α = 0.91 in the inpatient sample and α = 0.94 in the residential sample.

#### Fears of Compassion for Self (FCSelf; [5])

This 15-item self-report questionnaire measures fears of, or barriers to, self-compassion. Sample items include: “I fear that if I become kinder and less self-critical to myself then my standards will drop,” and “I feel that I don't deserve to be kind and forgiving to myself.” The FCSelf global score has demonstrated good internal consistency and convergent validity in a non-clinical sample [5] and has been shown to have a two-factor structure in individuals with EDs [8]. The Meeting Standards subscale (seven items) assesses concerns related to standards dropping, flaws showing, and fear that developing self-compassion would result in loss of relationships. The Emotional Vulnerability subscale (eight items) assesses deeper feelings of grief, avoidance of self-compassion, or feeling undeserving of self-compassion. The internal consistency of the FCSelf in the inpatient sample was α = 0.90 for the Meeting Standards subscale, and α = 0.84 for the Emotional Vulnerability subscale. The internal consistency of the FCSelf in the residential sample was α = 0.92 for the Meeting Standards subscale, and α = 0.85 for the and Emotional Vulnerability subscale. Consistent with previous research, in this study, the Pearson *r* correlation between the Meeting Standards and Emotional Vulnerability subscales were 0.61 and 0.65 in the inpatient and residential treatment samples, respectively.

#### Eating Disorders Inventory-3 (EDI-3; [17])

The EDI-3 is a 91-item measure that assesses psychological traits and symptoms relevant to the development and maintenance of an ED. This measure yields a total symptom score as well as four interpersonal/affective functioning scores. The total symptom score is a composite of items assessing drive for thinness, bulimia, and body dissatisfaction. The interpersonal problems (IP) scale assesses the extent to which social interactions and relationships are experienced as unrewarding, unsatisfying and/or artificial. The affective problems (AP) scale measures the ability to recognize and control emotional states. The over-control (OC) scale assesses beliefs that self-denial and self-sacrifice are virtuous. Finally, the ineffectiveness (IC) scale assesses insight about one’s affective states and feelings of self-worth. The five composite scores were used in the present research. Internal consistencies for the inpatient sample were α = 0.91 (EDS), 0.86 (IP), 0.85 (AP), 0.85 (OC), and 0.90 (IC). Internal consistencies for the residential sample were α = 0.90 (EDS), 0.71 (IP), 0.82 (AP), 0.80 (OC), and 0.82 (IC).

#### The Readiness and Motivation Questionnaire (RMQ; [18])

The RMQ assesses readiness to change ED symptoms. RMQ questions are used in conjunction with each of the 12 diagnostic questions from the Eating Disorder Examination [19], so that readiness information is obtained for each symptom. The RMQ yields total scores (averaged across all 12 symptoms) and symptom specific scores for the following motivational categories: Precontemplation, Action, Internality, and Confidence. All four total scores were used in this research. The RMQ has demonstrated good convergent, discriminant, and criterion validity in a clinical ED sample [18].

#### Eating Disorder Quality of Life Scale (EDQLS; [20])

This 40-item self-report measure is designed to assess quality of life in adults with EDs. The EDQLS provides a total quality of life (EDQLS total) score based upon twelve domains: school/work, relationships with others, feelings, leisure, thinking and concentrating, psychological health, family and close relationships, future, appearance, values and beliefs, general physical health, and health related to food and weight. The EDQLS has demonstrated excellent convergent validity and reliability in a diverse sample of college women [21]. The internal consistency of the EDQLS was α = 0.94 in the inpatient sample and α = 0.84 in the residential sample.

### Analysis plan

As this is a preliminary investigation of the potential role of self-compassion and barriers to self-compassion in predicting ED treatment outcomes, all analyses were considered exploratory in nature, and a liberal alpha of *p* < 0.05 was used to determine statistical significance. Given that this research is a first step to understanding the role of self-compassion and barriers to self-compassion to clinical outcomes, separate hierarchical linear regression analyses examined whether self-compassion and the two barriers to self-compassion were associated with each of the clinical outcome variables within each treatment setting.

For each self-compassion regression, the pre-treatment value of the outcome variable was entered in the first step and the total self-compassion score was entered in the second step. For each barrier regression, the pre-treatment value of the outcome variable was entered in the first step and the two barrier subscale scores were entered in the second step. We recognize the risk of Type 1 error, given the proposed number of regressions, with two families of analyses (one for self-compassion and one for barriers to self-compassion), conducted to examine the predictive utility of ten outcome variables in each sample (i.e., 20 analyses per family). At an alpha level of *p* < 0.05, we would anticipate 1/20 regressions in each family to be significant due to chance. In interpreting these exploratory findings, a large number of significant regressions would be required in order to have confidence in the predictive utility of either investigated variable (self-compassion or barriers to self-compassion).

## Results

Table 1 displays means, standard deviations, and effect sizes of pre-post treatment comparisons for clinical outcome variables for each treatment program. Table 2 displays means, standard deviations, and effect sizes of pre-post comparisons for the Self-Compassion Scale and Fears of Compassion for Self barrier subscale scores (Meeting Standards and Emotional Vulnerability) at pre and post for each treatment program.

**Table 1:** Description of study outcome variables at pre- and post-treatment

	Inpatient treatment (*n* = 87)			Residential treatment (*n* = 68)		
	Pre M (*SD*)	Post M (*SD*)	d*	Pre M (*SD*)	Post M (*SD*)	d*
Eating Disorder Inventory-3						
Total Symptoms	64.80 (20.67)	57.55 (20.88)	0.44*	58.14 (19.63)	44.38 (20.43)	0.76*
Drive for Thinness	20.47 (7.10)	18.41 (7.62)	0.36*	18.14 (5.82)	15.06 (7.43)	0.46*
Body Dissatisfaction	29.60 (10.00)	28.89 (10.44)	0.10	25.64 (10.17)	23.47 (10.09)	0.23
Bulimia	13.25 (10.15)	8.89 (8.34)	0.55*	14.25 (8.45)	6.74 (6.49)	1.19*
Interpersonal Problems	25.01 (10.19)	20.84 (9.60)	0.60*	20.67 (8.06)	16.90 (9.66)	0.45*
Affective Problems	27.04 (11.41)	23.32 (12.11)	0.48*	27.55 (12.44)	17.62 (11.58)	0.84*
Over control	28.10 (10.76)	24.70 (10.97)	0.41*	25.05 (10.54)	19.98 (10.16)	0.50*
Ineffectiveness	30.51 (11.18)	24.71 (11.59)	0.60*	24.70 (10.78)	19.08 (11.14)	0.45*
Readiness and Motivation						
Precontemplation	64.31 (18.61)	48.39 (21.23)	0.83*	53.96 (17.95)	35.15 (16.58)	0.99*
Action	40.23 (20.32)	68.01 (15.82)	− 1.32*	58.56 (20.92)	73.77 (16.58)	− 0.71*
Confidence	39.32 (18.83)	52.94 (20.12)	− 0.65*	50.22 (21.87)	67.74 (18.34)	− 0.96*
Internality	66.15 (22.38)	72.60 (19.82)	− 0.33*	75.98 (21.48)	85.74 (16.06)	− 0.55*
Quality of Life	91.60 (22.11)	112.34 (28.55)	− 0.91	110.86 (16.51)	132.49 (26.34)	− 0.84

*Significant at *p* < 0.001

**Table 2:** Description of Self-Compassion Scale and Fears of Compassion for Self barrier subscale scores at pre- and post-treatment

	Inpatient treatment (*n* = 87)			Residential treatment (*n* = 68)		
	Pre M (*SD*)	Post M (*SD*)	d*	Pre M (*SD*)	Post M (*SD*)	d*
Self-Compassion Scale	2.41 (0.72)	2.61 (0.71)	− 0.35	2.27 (0.70)	2.89 (0.79)	− 0.83
Barriers scores						
Meeting Standards	15.16 (8.05)	13.26 (8.50)	0.32	13.28 (7.87)	9.20 (8.57)	0.54
Emotional Vulnerability	18.93 (6.78)	15.79 (7.79)	0.57	14.95 (7.06)	11.37 (7.40)	0.44

*All effect sizes were significant at *p* < 0.001

### Regression analyses

#### Self-compassion

Pre-treatment self-compassion scores contributed to the prediction of post-treatment outcomes in 2/20 regressions. Specifically, higher levels of pre-treatment self-compassion were associated with greater improvements in EDI-3 ineffectiveness in both the inpatient, Δ*R*^2^ = 0.02, *F*(2,83) = 44.59, *p* < 0.01 and residential samples, Δ*R*^2^ = 0.08, *F*(2,58) = 5.38, *p* < 0.01.

#### Barriers to self-compassion

Barrier subscale scores on the Fears of Compassion for Self scale contributed significantly to the prediction of post-treatment outcomes in 15/20 regressions: seven from the inpatient sample and eight from the residential sample. As shown in Table 3, across both patient samples, pre-treatment Emotional Vulnerability barriers accounted for additional variance in the four EDI-3 interpersonal/affective functioning scores and EDQLS quality of life scores at post-treatment. Pre-treatment Meeting Standards barriers accounted for additional variance in post-treatment RMQ readiness scores. Of note, post-treatment ED symptoms were associated with a different pre-treatment barrier depending upon the population; fewer Meeting Standards barriers were associated with greater ED symptom reduction in inpatient treatment, whereas fewer Emotional Vulnerability barriers were associated with greater ED symptom reduction in residential treatment.

**Table 3:** Significant hierarchical multiple regression models predicting post-treatment outcome variables from pre-treatment Fears of Compassion for Self barrier subscale scores (Meeting Standards and Emotional Vulnerability) in Inpatient and Residential Treatment

		*R*^2^	*F*	*B*
EDI-3 Eating Disorder Symptoms				
Inpatient				
Step 1	Pre-treatment EDI-3 Total Symptoms	0.62	132.46***	0.79***
Step 2	Meeting Standards	0.66	77.16***	0.22**
Residential				
Step 1	Pre-treatment EDI-3 Total Symptoms	0.31	25.14***	0.55***
Step 2	Emotional Vulnerability	0.42	20.11***	0.37**

		*R*^2^	*F*	*B*
EDI-3 Interpersonal Problems				
Inpatient				
Step 1	Pre-treatment EDI-3 IP	0.62	138.99***	0.79***
Step 2	Emotional Vulnerability	0.65	77.25***	0.20**
Residential				
Step 1	Pre-treatment EDI-3 IP	0.32	27.31***	0.57***
Step 2	Emotional Vulnerability	0.39	17.83***	0.28*

		*R*^2^	*F*	*B*
EDI-3 Affective Problems				
Residential				
Step 1	Pre-treatment EDI-3 AP	0.21	14.20***	0.45***
Step 2	Emotional Vulnerability	0.28	10.69***	0.35*

		*R*^2^	*F*	*B*
EDI-3 Overcontrol				
Residential				
Step 1	Pre-treatment EDI-3 OC	0.20	13.78***	0.45***
Step 2	Emotional Vulnerability	0.32	12.59***	0.38**

		*R*^2^	*F*	*B*
EDI-3 Ineffectiveness				
Inpatient				
Step 1	Pre-treatment EDI-3 IC	0.49	82.00***	0.70***
Step 2	Emotional Vulnerability	0.53	47.58***	0.26**
Residential				
Step 1	Pre-treatment EDI-3 IC	0.08	5.19*	0.28*
Step 2	Emotional Vulnerability	0.22	8.24***	0.40**

		*R*^2^	*F*	*B*
RMQ Precontemplation				
Inpatient				
Step 1	Pre-treatment RMQ Precon	0.30	25.49***	0.55***
Step 2	Meeting Standards	0.40	19.90***	0.34**

		*R*^2^	*F*	*B*
RMQ Action				
Inpatient				
Step 1	Pre-treatment RMQ Action	0.21	15.99***	0.46***
Step 2	Meeting Standards	0.27	10.91***	− 0.24*
Residential				
Step 1	Pre-treatment RMQ Action	0.15	11.36***	0.39***
Step 2	Meeting Standards	0.23	9.12***	− 0.27*

		*R*^2^	*F*	*B*
RMQ Confidence				
Inpatient				
Step 1	Pre-treatment RMQ Confidence	0.26	20.64***	0.51***
Step 2	Meeting Standards	0.36	16.67***	− 0.33**
Residential				
Step 1	Pre-treatment RMQ Confidence	0.39	40.95***	0.63***
Step 2	Meeting Standards	0.43	23.72***	− 0.20*

		*R*^2^	*F*	*B*
Eating Disorder Quality of Life				
Inpatient				
Step 1	Pre-treatment Quality of Life	0.49	67.23***	0.70***
Step 2	Emotional Vulnerability	0.54	39.60***	− 0.26*
Residential				
Step 1	Pre-treatment Quality of Life	0.12	9.20**	0.35**
Step 2	Emotional Vulnerability	0.21	8.60***	− 0.32**

*EDI-3* Eating Disorders Inventory-3, *IP* interpersonal problems, *AP* affective problems, *OC* overcontrol, *IC* ineffectiveness, *RMQ* Readiness and Motivation Questionnaire

*p < 0.05, **p < 0.01, ***p < 0.001

## Discussion

This exploratory research examined the importance of self-compassion and two barriers to self-compassion in predicting a range of clinical outcome variables for patients undergoing inpatient and residential ED treatment. The large number of significant regressions for barriers to self-compassion (15/20) suggest that the relation between barriers and outcome is not due to chance. Findings also suggest that barriers to self-compassion play a more consistent role than self-compassion (where only 2/20 regressions were significant). Given that few reliable predictors of treatment outcome have been established in individuals with EDs [13, 14], it is noteworthy that this is the second study (in addition to 9) to have found that barriers to self-compassion predict treatment outcomes in patients with EDs, and that in the present study, the two barriers types, Meeting Standards and Emotional Vulnerability, both emerged as uniquely relevant.

In both residential and inpatient samples, having fewer Emotional Vulnerability barriers to self-compassion was associated with greater improvements in interpersonal problems, affective problems, overcontrol, ineffectiveness, and quality of life. In the development of self-compassion, feeling deserving of self-kindness, and being open to one’s emotional experience may make it possible to learn and practice affect regulation skills. These skills in turn have been associated with favourable outcomes in ED treatment [22]. Given the breadth of domains associated with Emotional Vulnerability barriers found in this study, it may be beneficial to specifically target these in treatment. Acknowledging the challenge of experiencing difficult emotions, providing a safe space for patients to connect with their feelings, providing validation regarding traumatic or harmful experiences that may make self-compassion challenging, and modelling a self-compassionate attitude may help. In a recent qualitative study, patient participants described validation from staff, family members and other patients as central to their ability to overcome Emotional Vulnerability barriers [23]. Therefore, it may be beneficial for patients to be in the presence of others, such as in a group environment where they are able to witness peers benefitting from working through difficult emotions. This may facilitate trust and encourage openness to explore self-compassion as a tool for addressing one’s own emotions. Indeed, preliminary research suggests that hearing a peer cope self-compassionately with body image distress can facilitate increased self-compassion vis-à-vis personal body image distress in the listener [24].

In both samples, Meeting Standards barriers were associated with fewer improvements in readiness and motivation to change one’s ED. These relations may be explained by links among Meeting Standards barriers, readiness, and identity. That is, identity may be experienced as a reflection of one’s achievements, including shape and weight. Thus, for both Meeting Standards barriers and readiness for change, patients may be confronted with the question, “Who am I in the absence of my accomplishments, some of which may be tied to my ED?” For individuals with these barriers, engaging in self-compassion practices may feel akin to losing one’s drive or becoming lazy, and hence represent a threat to a valued way of viewing oneself. One way in which it may be helpful to address these fears is by informing patients that greater self-compassion is not associated with one’s standards or achievements dropping, but rather increases one’s motivation to self-improve in a variety of domains [25]. Providing and reinforcing this evidence-based psychoeducation, while also encouraging patients to engage in behavioural experiments with self-compassion to test their assumptions for themselves, may be effective in reducing this barrier.

Interestingly, pre-treatment self-compassion barriers were also predictive of ED symptom change, but the relevant barrier differed by treatment setting. Lower Meeting Standards barriers were associated with ED symptom improvement in inpatient treatment, while lower Emotional Vulnerability barriers were associated with improvements in ED symptoms in the residential treatment program. This may be a function of the different presentations of patients accessing these two levels of care, as was observed in this research. Canadian clinical practice guidelines recommend that inpatient treatment focus on medical stabilization and symptom interruption, and is typically offered to patients who have lower levels of readiness for change, higher levels of ED and psychiatric symptomatology, and who are more likely to have a diagnosis of anorexia nervosa [16]. In contrast, day or residential treatment is recommended for patients with higher levels of readiness and focuses on recovery [16]. Possibly, for individuals at earlier stages of recovery in which weight gain and/or identity fears are at the forefront, Meeting Standards concerns are most prominent and improvements in ED symptoms are facilitated by lower levels of this type of barrier. In contrast, for individuals choosing to enroll in treatment focusing on full recovery, willingness to address the emotional underpinnings of the ED may be most critical to meeting the different goals of maintaining a healthy lifestyle and practicing coping tools as an alternative to ED behaviours.

Unlike barriers to self-compassion, self-compassion was not consistently associated with outcome in either treatment setting. This is perhaps not surprising given that few predictors of outcome have been detected in individuals with EDs [13, 14]. As such, the lack of relation between self-compassion and outcome is perhaps less noteworthy than the consistency of relations between self-compassion barriers and outcome. Possibly, barrier scores are a proxy to low readiness. As noted earlier, when readiness for behavioural change was found to be a predictor of outcome in individuals with EDs, treatments were enhanced by directly assessing and addressing readiness and ensuring that treatment was matched to patient needs [18]. If barriers are associated with outcome because they are a reflection of low readiness to cultivate self-compassion, it may similarly be helpful to develop a more direct measure of readiness for self-compassion, and target this early on in future treatment.

A limitation of this study is that it was conducted with patients attending intensive hospital-based ED treatment and the generalizability of findings to less acute ED populations is not known. In addition, we did not have a sufficient sample size to conduct analyses on diagnostic subgroups. Finally, given that this is the first longitudinal study to investigate the relation between these two barriers to self-compassion and symptom change in individuals with EDs, these findings require replication.

## Conclusions

Findings from this research suggest that the benefits individuals with EDs experience from treatment may be enhanced by assessing and addressing the unique types of barriers to self-compassion they face. Increasing awareness that patients may vary in their readiness for self-compassion could be a first step in lowering these barriers and improving outcomes of ED treatment. This research suggests that addressing Meeting Standards barriers may be particularly helpful for individuals with more acute symptoms and lower readiness for change, and that Emotional Vulnerability barriers may play a more important role for individuals who are less acutely ill, with higher readiness for change. Future research could explore the best ways of assessing readiness for self-compassion in the EDs and whether a consideration of the specific type(s) of barriers a patient faces—concerns about Meeting Standards and/or concerns about emotional vulnerability—can inform more effective and sensitive clinical approaches. It would also be fruitful to develop and test possible interventions, such as ones that help patients explore the perceived positive and negative functions of barriers, which may ultimately help patients decide that a more self-compassionate stance is worth adopting.

## Data Availability

The data that support the findings of this study are available from the corresponding author upon reasonable request.

## Abbreviations

ED: Eating disorder
FCSelf: Fears of Compassion for Self scale
SCS: Self-Compassion Scale
EDI-3: Eating Disorders Inventory-3
IP: Interpersonal problems
AP: Affective problems
OC: Overcontrol
IC: Ineffectiveness
RMQ: Readiness and Motivation Questionnaire
EDQLS: Eating Disorders Quality of Life Scale
